# Pigs’ capacity to experience feelings and to suffer from tail lesion, ear lesion and lameness: Exploring citizens and pig farm and abattoir workers’ knowledge and perceptions

**DOI:** 10.1371/journal.pone.0286188

**Published:** 2023-05-25

**Authors:** Dayane Lemos Teixeira, Laura C. Salazar, Daniel Enriquez-Hidalgo, Maria José Hötzel

**Affiliations:** 1 Department of Animal and Agriculture, Hartpury University, Gloucester, United Kingdom; 2 Instituto de Ciencias Agroalimentarias, Animales y Ambientales (ICA3), Universidad de O’Higgins, San Fernando, Chile; 3 Departamento de Ciencias Animales, Pontificia Universidad Católica de Chile, Santiago, Chile; 4 Bristol Veterinary School, University of Bristol, Langford, United Kingdom; 5 Laboratório de Etologia Aplicada e Bem-Estar Animal, Departamento de Zootecnia e Desenvolvimento Rural, Universidade Federal de Santa Catarina, Florianópolis, Brazil; Universidade Federal de Minas Gerais, BRAZIL

## Abstract

The aim of this study was to gain insight into the perceptions of pig farm and abattoir workers as well as lay citizens regarding (1) sentience and (2) positive (intelligent and friendly) and negative (gluttonous, stubborn and dirty) attributes of pigs. We also aimed to investigate the (3) knowledge and perceptions of pig farm and abattoir workers on tail lesion, ear lesion and lameness in pigs and (4) the opinion of lay citizens regarding the likelihood of tail lesions, ear lesions, and lameness causing suffering in pigs and affecting meat quality. Chilean pig farm workers (n = 116), pig abattoir workers (n = 95), and lay citizens (n = 708) were invited on farm, at the abattoir and in public places, respectively, to participate in a survey. Answers were indicated using a 5-point Likert scale (0 = totally disagree; 4 = totally agree). Data were analysed using generalized linear models, including recruitment place and socio-demographic data as predictor variables. Female and lay citizens attributed pigs a higher capacity to experience feelings than male participants and pig farm and abattoir workers (p < 0.05). Lay citizens and workers recruited on farm described pigs as being more intelligent and friendly than those workers recruited at the abattoir (p < 0.001); recruitment place and sex were not associated with participants’ perception regarding negative attributes of pigs (p > 0.05). Most lay citizens considered that tail lesions, ear lesions and lameness are likely to cause suffering in pigs and older participants had higher odds of agreeing that tail and ear lesions are likely to affect meat quality (p < 0.05). Finally, the risk factors for tail lesion, ear lesions and lameness pointed out by pig farm and abattoir workers is in line with what has been suggested by experts. Our findings contribute to understand the perception and values of all stakeholders regarding animal welfare, as it is crucial to improve the sustainability of animal production systems.

## Introduction

An animal is in a good state of welfare if it is healthy, comfortable, well nourished, safe, able to express innate behaviours, and if it is not suffering from unpleasant states, such as pain, fear, and distress [1]. But, does everyone have the same perception about the capacity of animals to experience feelings? More specifically, does the perception towards animal sentience differ between pig farm and abattoir workers and lay citizens? Gaining knowledge on the attitudes of all stakeholders is crucial to improve animal welfare and thus the sustainability of animal production systems [2].

Lay citizens in general believe that animals are sentient, with the capacity to have emotional states and to suffer (e.g. [3, 4]) and consider that imposing pain on animals is unacceptable (e.g. [5, 6]). Citizens also tend to reject intensive animal production systems and practices that they believe cause stress and suffering to animals, and prefer systems where animals can have positive emotional states, which they often associate with higher quality products [7, 8]. Citizens’ and consumers’ interest about the living conditions and welfare of food producing animals is increasing, which has led to the development and significant changes in legislations and market led initiatives regarding the care of farm animals [9, 10]. Given the importance attributed by citizens and consumers to husbandry practices that impact positively or negatively on the emotional states in farm animal [11–13], these practices are increasingly considered in these legislations and initiatives.

However, the perception of stakeholders involved in the animal industries regarding animal sentience and welfare has been less investigated. Knowing farmers’ and animal caretakers’ views and perceptions about animal sentience and welfare is important, given that these stakeholders are considered directly responsible for the welfare of the animals under their care. Many stakeholders directly involved in the pig industry are responsible for animal husbandry, which in the pig industry includes the practice of painful procedures such as tail docking and castration in some countries. It is common to think that farmers cope with potential negative impacts of farming practices on animals because, in contrast with people not involved in animal production, they ascribe low capacity to suffer and feel emotions to their animals. However, this may not be true, at least in the pig industry. For example, pig farmers recognize that castration, tail docking, ear notching and parturition are painful for pigs [14–16]. Also, a recent study found that pig farmers expressed similar beliefs in pigs’ capacity to suffer as citizens [3].

Recognising sentience may improve attitudes towards animal welfare and intentions to improve it [17]. However, to make decisions to improve animal welfare also requires that the people in charge of caring for the animals recognize indicators of animal welfare and the underlying causes of animal welfare outcomes. Previous studies suggested that different risk factors on farm and during transport might play a role on animal-based welfare outcomes, due to the wide variation in the prevalence among pens on farm and among batches at the abattoir [18–20]. Tail and ear lesions are multifactorial in nature and seems to share similar risk factors [21], such as low quality of bedding material, high stocking density, unbalanced dietary and poor ventilation [22–24]. Also, floor type, nutrition are stocking density are common risk factors associated with lameness in pigs [25–29]. However, much research on risk factors related to animal-based welfare outcomes has been done in controlled environmental conditions and small sample sizes. Additionally, the awareness of stakeholders involved in pig production regarding these aspects has not been investigated before.

The aim of this study was to gain insight into the perceptions of pig farm and abattoir workers as well as lay citizens regarding (1) sentience and (2) positive (intelligent and friendly) and negative (gluttonous, stubborn and dirty) attributes of pigs. We also aimed to investigate the (3) knowledge and perceptions of pig farm and abattoir workers on tail lesion, ear lesion and lameness in pigs and (4) the opinion of lay citizens regarding the likelihood of tail lesions, ear lesions, and lameness causing suffering in pigs and affecting meat quality. We hypothesized that, compared to lay citizens, pig farm and abattoir workers would attribute pigs less capacity of pigs to experience emotions and would describe pigs as being more gluttonous, stubborn and dirty; we also hypothesized that pig farm and abattoir workers would be aware about the underlying causes of animal welfare outcomes on farm, and that lay citizens would believe that welfare outcomes cause suffering in pigs and affect meat quality.

## Materials and methods

### Participant recruitment

The Scientific Ethic Committee for Animals and Environmental Care of the Pontifícia Universidad Católica de Chile (protocol number 170529006) and by the Research Department Ethic Committee of the Universidad de O’Higgins (No. 002–2020) approved the research project and the survey. The consent form was exempt for the recruitment in public places.

This study was carried out in Chile between August 2018 and August 2019 and consisted of a survey with 947 participants. The survey used face-to-face questionnaire and participants were recruited by personal invitation in different places: 5 commercial large pig farms, 1 commercial pig abattoir and public places such as parks, medical clinic waiting areas, bus station and shopping malls, all places where people were waiting or had free time. Only people that were at least 18 years old were included in the study and their identity was not required. On farms and at the abattoir, the questionnaire was carried out during shift breaks, lunch times and at the end of working days, and all workers was personally invited, independently of their occupation at the farm. In these places, two workers were not able to self-read the questionnaire, as they were illiterate or semi-literate, and requested assistance to the recruiter. Due to a confidential agreement between the authors and the company, further information about the farm and the abattoir are not included in the manuscript.

The first 20 responses of the questionnaire carried out on farm and at public places were conducted as a pilot study, and answers and comments were discussed among the research team to refine the final version of the questionnaire. Only small amendments were needed for the final version of the questionnaire, and these first 20 responses were discarded to avoid affecting the results.

All participants were asked if they would like to participate in a survey about pig production, without any other specification. After acceptance, participants from the farms and the abattoir were invited to read a consent form and sign it before taking the survey. The Ethic Committees that approved the study exempted the need of a consent form for the questionnaires applied in public places. All data collected in the three set of places (farms, abattoir, public places) were transcribed to a Google Form and all information was automatically transcribed to a Microsoft® Excel sheet for Mac 2011.

### Description of the survey

The questionnaire had three parts: **Part 1**—participant socio-demographic information; **Part 2**—Perception of pig farm and abattoir workers and lay citizens regarding sentience and positive and negative attributes of pigs; **Part 3**—Knowledge and perceptions of pig farm and abattoir workers on tail lesion, ear lesion and lameness in pigs and lay citizen opinion regarding the likelihood of tail lesion, ear lesion and lameness cause suffering in pigs and that they affect meat quality.

The questionnaire was identical for participants recruited on farm and at the abattoir, however the questions included in Part 3 were different for participant recruited in public places.

#### Part 1

*Socio-demographic information*. The first questions addressed participants’ socio-demographic information relating to sex (male; female), age (18–25; 26–35; 36–45; 46–55; over 56 years old), education level (up to high school; higher education—completed or on-going). Participants from the farms and the abattoir were asked to specify the working place where they carry out their duties (later grouped as: administration/office; field work—animal/carcass handling; animal transportation—truck driver); participants invited in public places were asked their involvement in agriculture (not involved; professional involvement–rural producer, student, academic, etc.; not currently involved but grew up in an agricultural environment) and their consumption pattern in relation to animal products [not meat consumers, meat consumers (beef, pork and/or poultry)].

#### Part 2

*Perception of pig farm and abattoir workers and lay citizens regarding sentience and positive and negative attributes of pigs*. All participants were then asked their level of agreement with the capacity of pigs to experience feelings as pain, fear, happiness, anxiety and boredom; and their level of agreement that pigs have the following attributes: they are intelligent, gluttonous, friendly, stubborn and dirty. Answers were indicated using a 5-point Likert scale (0 = totally disagree; 4 = totally agree). Participants could also answer ‘do not know’.

#### Part 3

*Knowledge and perceptions of pig farm and abattoir workers on tail lesion*, *ear lesion and lameness in pigs*. Participants recruited on farm and at the abattoir were asked how they considered the prevalence (number of cases) of each welfare outcome (tail lesion, ear lesion and lameness) in pigs from the farm/abattoir where they worked [options were: does not exist (0%); low (0–25%); neither low nor high (25–50%); high (50–75%); very high (75–100%); do not know]; and what they considered to be the main reasons for the presence of each of these conditions in pigs [options were: management; lack of environmental enrichment; use of antibiotics; diet; animal sex; animal density; floor type; number of pen/building; thermal insulation type; pen orientation (south/north); pen localization within building; others; do not know]. These reasons were selected because this survey was part of a broader study evaluating animal-based welfare outcomes in slaughter pigs on farm and at the abattoir, including assessing the association with some potential risk factors and the presence of ear lesions, tail lesions and lameness on farm (unpublished data).

*Lay citizen opinion regarding the likelihood of tail lesion*, *ear lesion and lameness cause suffering in pigs and that they affect meat quality*. Participants recruited in public places were asked to rate the likelihood of tail lesions, ear lesions, and lameness causing suffering in pigs, and to rate the likelihood of these conditions to affect the meat quality (flavour, odour, colour and/or other aspect of the meat). Options were unlikely, neutral and likely. Participants could also answer ‘do not know’.

### Statistical analysis

Descriptive statistics for the responses were calculated using Microsoft® Excel and all other statistical analyses were conducted using SAS 9.3. Participants that did not answered all closed questions related to socio-demographic information (sex, age, level of education; n = 28) were excluded; therefore 919 usable questionnaires were analysed. Due to the low number of participants in these categories, professional involvement and those not currently involved but grew up in an agricultural environment were grouped as ‘involved in agriculture’.

In **Part 2**, consistency of the five questions regarding pig sentience (i.e., how much they agree with the capacity of pigs to experience feelings as pain, fear, happiness, anxiety and boredom), consistency of the two questions regarding the positive attributes towards pigs (i.e. how much they agree that pigs are intelligent and friendly) and consistency of the three questions regarding the negative attributes towards pigs (i.e. how much they agree that pigs are gluttonous, stubborn and dirty) were assessed using Spearman correlation coefficients (PROC CORR). Answers to the capacity of pigs to experience feelings as pain, fear, happiness, anxiety and boredom were correlated (ρ > 0.530; p < 0.001) so these responses were averaged to create a mean for each participant for their “**perception regarding the capacity of pigs to experience feelings**”. Answers regarding pigs being intelligent and friendly were correlated (ρ = 0.602; p < 0.001) so these responses were averaged to create a mean for each participant for their “**perception regarding positive attributes towards pigs**”. Answers regarding pigs being gluttonous, stubborn and dirty were correlated (ρ > 0.388; p < 0.001) so these responses were averaged to create a mean for each participant for their “**perception regarding negative attributes towards pigs**”. After excluding participants that left at least one closed question unanswered or answered do not know in any perception questions, the final number of participants included in the Spearman correlation analysis were 900, 779 and 753, respectively. The effects of the survey recruitment place (farm, abattoir, public places) and the social-demographic questions (sex, age and level of education) on participant perceptions were tested using generalized linear models (PROC GLIMMIX), including recruitment place, sex, age and level of education as independent variables, participant perceptions as dependent variables, gamma as distribution and glogit link function. Interactions between recruitment place and socio-demographic information was not included due to the low number of participants in different categories. Post-hoc comparisons were performed using a Tukey HSD test. Statistics associations were reported when p < 0.05 and tendency when 0.05 < p < 0.1.

In **Part 3**, generalized linear models (PROC GLIMMIX) were used to analyse associations between the socio-demographic information of participants recruited in public places, including their involvement in agriculture and meat consumption pattern (independent variables), and their opinion regarding the likelihood of tail lesion, ear lesion and lameness causing suffering in pigs and the likelihood of these conditions affecting meat quality (dependent variables), including glogit link function. Participants that answered do not know in the opinion questions were excluded from this analysis. Univariate models were built to separately assess the influence of each predictor variable on the dependent variables. Predictor variables with P < 0.20 [30] were used to build multivariate models. Backward selection was used to eliminate predictor variables until only those with P < 0.10 remained in the models. Results are presented as odds ratio (OR) and 95% confidence interval (95% CI). Statistics associations were reported when P < 0.05 and tendency when 0.05 < P < 0.1.

## Results

### Part 1

#### Socio-demographic information

Socio-demographic information from participants recruited on farm, at the abattoir and in public places is shown in Table 1. In general, most participants completed up to high school. Also, most participants recruited on farm and at the abattoir were male and carried out their duties on the field, and most participants recruited in public places were not involved in agriculture and were meat consumers.

**Table 1:** Socio-demographic data (percentage) of the participants in each recruitment place (n = 919).

Participant information	Recruitment place			Total (n = 919)
	Farms (n = 116)	Abattoir (n = 95)	Public places (n = 708)	
**Sex (%)**				
Female	9	17	52	42
Male	91	83	48	57
**Age (%)**				
18–25	25	9	24	22
26–35	33	24	19	21
36–45	10	34	20	21
46–55	13	20	19	18
>56	9	30	18	16
**Level of education (%)**				
Up to high school	92	86	68	72
Higher education ^1^	8	14	32	28
**Working place (%)**				
Office	7	4	-	6
Field work	93	91	-	92
Animal transportation	0	5	-	2
**Involvement in agriculture (%)**				
Not involved	-	-	78	78
Involved	-	-	22	22
**Meat consumption patter (%)**				
Not consumer	-	-	14	14
Consumer	-	-	86	86

^1^ Graduated or on going.

### Part 2

#### Perception of pig farm and abattoir workers and lay citizens regarding sentience and positive and negative attributes of pigs

**Fig 1:**
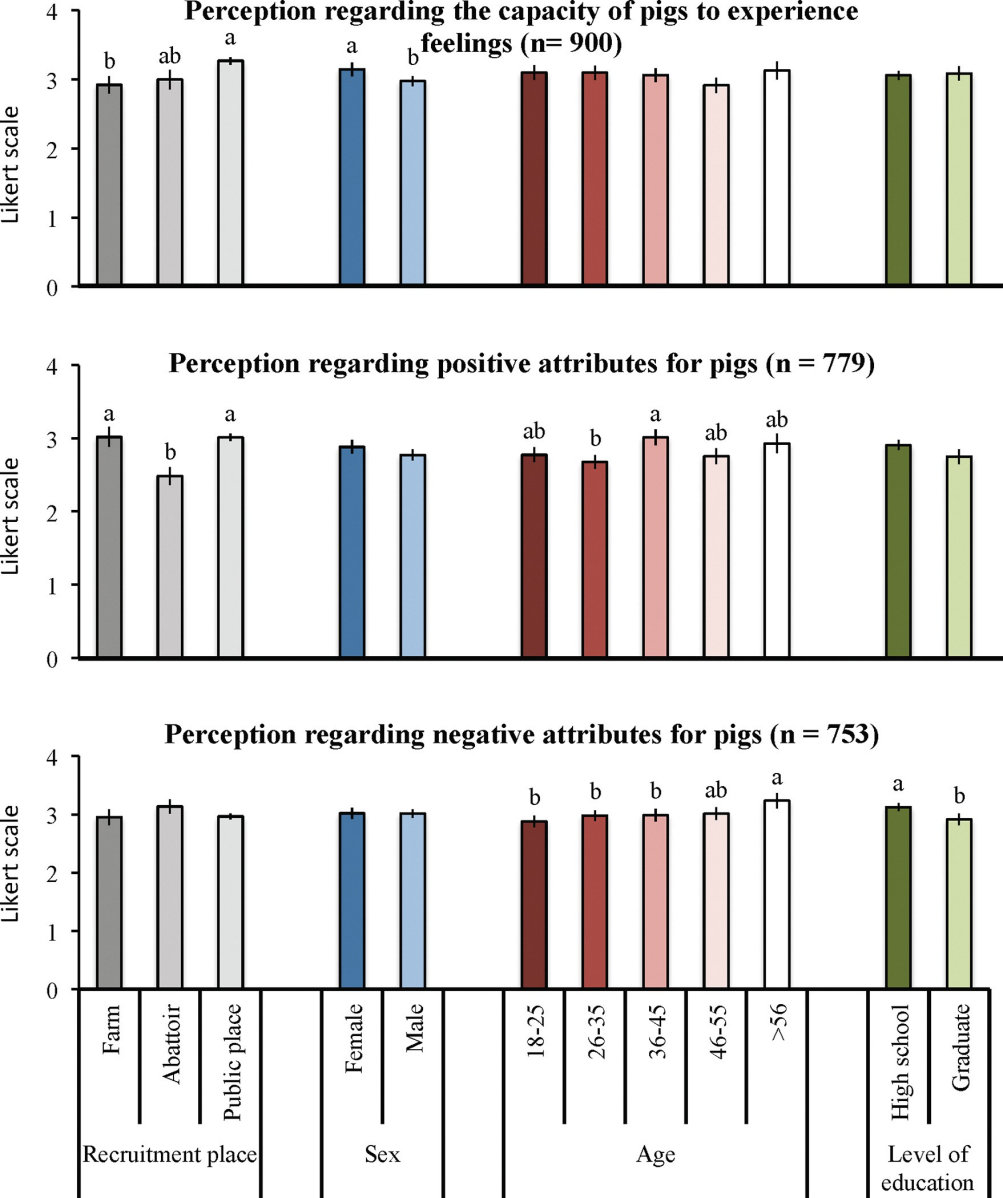
Participant perception regarding the capacity of pigs to experience feelings (pain, fear, happiness, anxiety and boredom) and regarding positive (intelligent, friendly) and negative attributes (gluttonous, stubborn, dirty) for pigs. Likert scales from 0 (totally disagree) to 4 (totally agree). Different letters indicate p < 0.05 within recruitment place and socio-demographic information.

Lay citizens attributed pigs with higher capacity to experience feelings (such as pain, fear, happiness, anxiety and boredom) than pig farm workers but not abattoir workers (p < 0.01; Fig 1); similarly, female participants attributed pigs with higher capacity to experience feelings than male participants (p < 0.05; Fig 1). Age and level of education did not affect the perception of participants regarding the capacity of pigs to experience feelings (p > 0.05; Fig 1).

Lay citizens and workers recruited on farm described pigs as being more intelligent and friendly than those workers recruited at the abattoir (p < 0.001). Similarly, participants who were 36–45 described pigs as being more intelligent and friendly than those who were 26–45 years old (p < 0.05; Fig 1). Sex and level of education did not affect participants’ perception regarding positive attributes of pigs (p > 0.05; Fig 1).

Participants older than 56 years old and participants with up to high school as level of education described pigs as being more gluttonous, stubborn and dirty than those between 18 and 45 years old (p < 0.05) and those with completed or on-going graduation (p < 0.05). Recruitment place and sex did not affect participants’ perception regarding negative attributes of pigs (p > 0.05; Fig 1).

### Part 3

#### Knowledge and perceptions of pig farm and abattoir workers on tail lesion, ear lesion and lameness in pigs

**Fig 2:**
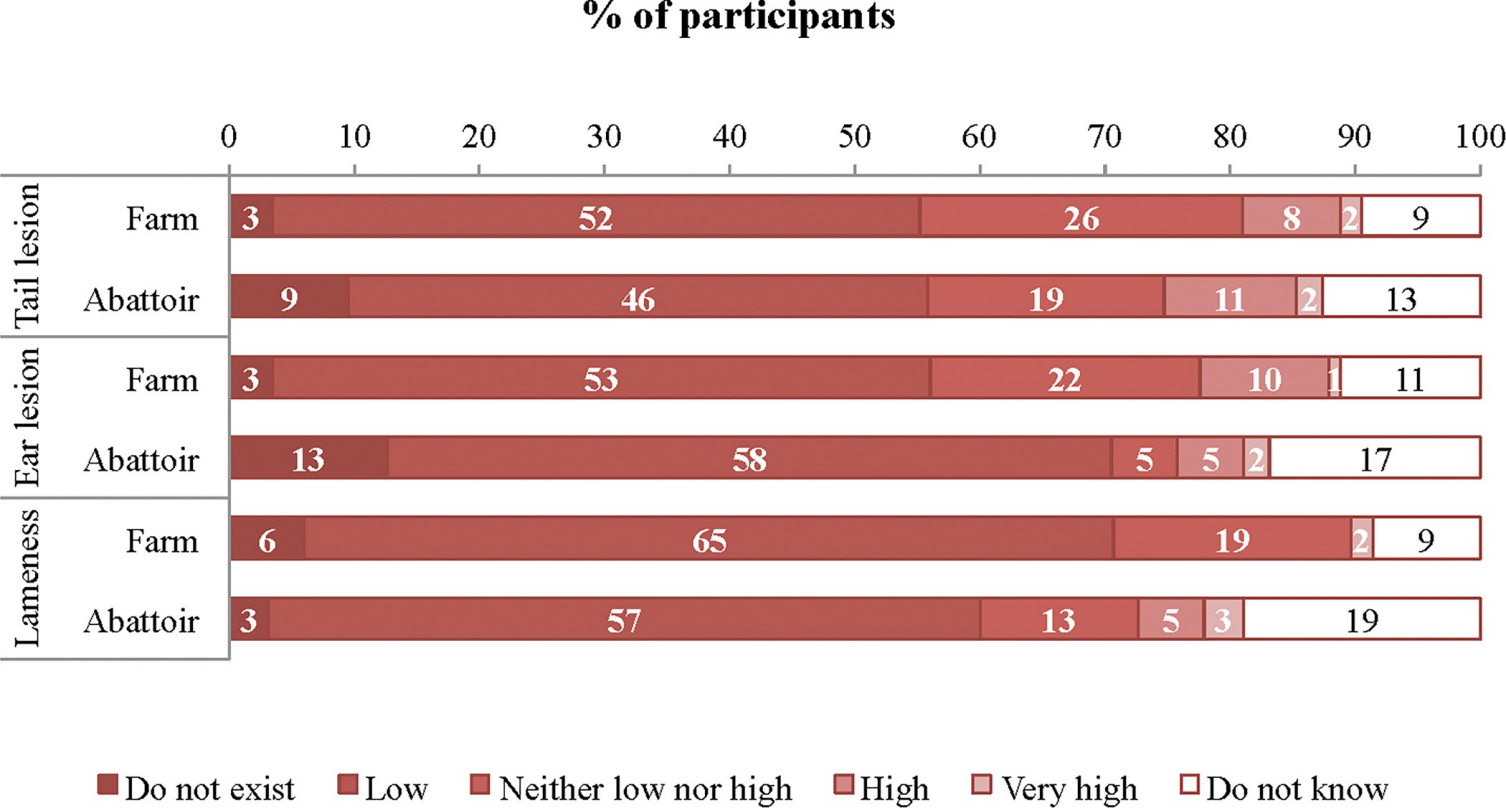
Pig farm (n = 116) and abattoir (n = 95) workers opinion regarding the prevalence of tail lesion, ear lesion and lameness in pigs from the farm/abattoir where they work.

Among workers recruited on farm and at the abattoir, most indicated that the prevalence of tail lesion, ear lesion and lameness in pigs from the farm/abattoir where they worked was low (Fig 2). Workers recruited in both places cited similar reasons for the presence of tail and ear lesions; the main reasons they cited for the presence of tail lesions were stocking density (farm: 24%; abattoir: 29%), diet (farm: 16%; abattoir: 20%), lack of environmental enrichment (farm: 16%; abattoir: 20%), and management (farm: 11%; abattoir: 14%). The main reasons cited for the presence of ear lesions were stocking density (farm: 22%; abattoir: 27%), diet (farm: 17%; abattoir: 21%), management (farm: 13%; abattoir: 16%), and lack of environmental enrichment (farm: 12%; abattoir: 15%). Management (farm: 32%; abattoir: 39%), type of floor (farm: 25%; abattoir: 31%), and stocking density (farm: 16%; abattoir: 19%) were the main reasons cited for lameness in pigs.

#### Lay citizen opinion regarding the likelihood of tail lesion, ear lesion and lameness cause suffering in pigs and that they affect meat quality

**Fig 3:**
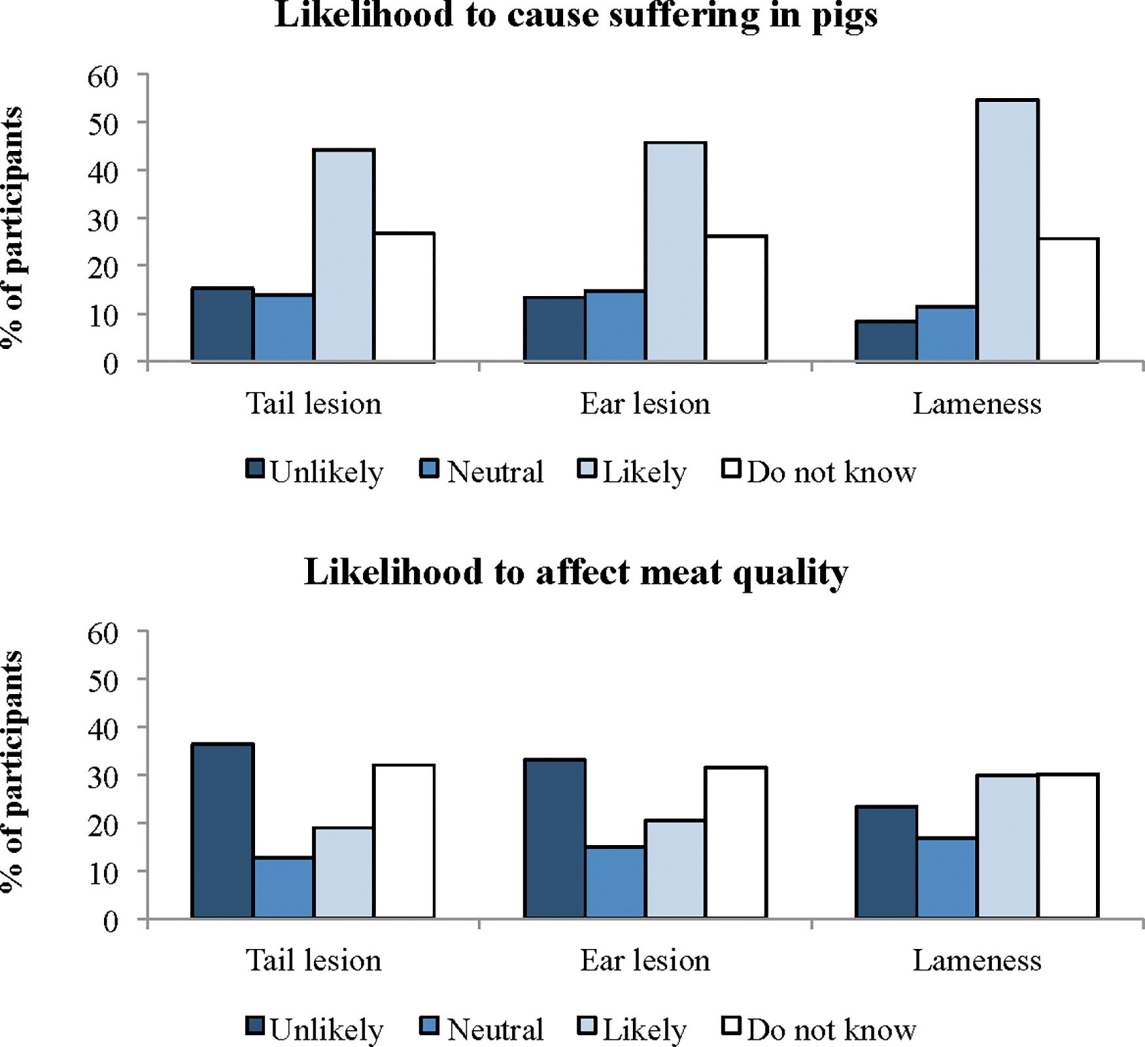
Lay citizen (n = 708) opinion regarding the likelihood of tail lesion, ear lesion and lameness cause suffering in pigs and that they affect meat quality.

Fig 3 shows that most lay citizens considered that tail lesions, ear lesions and lameness are likely to cause suffering in pigs. Participants who were older than 56 years old had higher odds of agreeing that tail lesions (OR = 2.3, 95% CI = 1.17–4.42) and ear lesions (OR = 2.6, 95% CI = 1.31–5.08) are likely to cause suffering in pigs than those who were 18–25 years old (P < 0.05). Male participants recruited in public places had higher odds of agreeing that ear lesions are unlikely to cause pig suffering than female participants (OR = 2.2, 95% CI = 1.36–3.72; P < 0.01). Lay citizens who were older than 56 years old had higher odds of agreeing that tail lesions (OR = 2.7, 95% CI = 1.28–6.05) and ear lesions (OR = 3.3, 95% CI = 1.64–6.76) are likely to affect meat quality (P < 0.05). In contrast, participants with up to high school as level of education had higher odds of agreeing that ear lesions are unlikely to affect meat quality (OR = 1.6, 95% CI = 1.01–2.49; P < 0.05). Finally, none of the independent variables affected participant opinion regarding the likelihood of lameness causing suffering in pigs and affecting meat quality (P > 0.05).

## Discussion

### Knowledge and perceptions of pig farm and abattoir workers

The majority of pig farm and abattoir workers indicated that the prevalence of tail lesion, ear lesion and lameness in pigs from the farm/abattoir where they worked were low. The perceived lameness prevalence is in line with the findings reported by Teixeira et al. [18], who assessed the prevalence of animal-based pig welfare outcomes on the same farm and abattoir where our participants were recruited. In contrast, Teixeira et al. [18] reported a higher prevalence of ear lesions on farm and tail lesion at the abattoir. However, it is important to note that the findings of Teixeira et al. [18] were based on the percentage of pens on farm and batches at the abattoir with at least one pig affected by tail and ear lesions, which differ from the current study, as participants were asked to estimate the prevalence of these conditions based in number of cases, regardless of pens or batches. Lay citizens considered that tail lesions, ear lesions and lameness are likely to cause suffering in pigs, thus a low prevalence of these welfare outcomes partially meets their expectation.

When given a choice, pig farm and abattoir workers pointed out stocking density, diet and lack of environmental enrichment as the main reasons for the presence of tail lesions, which is in line with what has been proposed by experts [31, 32]. The risk factors for ear lesion pointed out by our participants (stocking density, diet, management and lack of environmental enrichment) are also in line with what has been suggested by experts, as ear lesions seems to share similar risk factors than tail lesions [21]. However, there are no studies investigating environmental, housing and management conditions throughout all growing and fattening stages of pigs as risk factors for ear lesions in intensive pig production, thus further investigation evaluating these aspects would be beneficial for more explicit association. Finally, participants involved in pig production cited management, type of floor and stocking density as the main reasons for lameness in pigs, which is also in line with previous studies evaluating these conditions in pigs in different growing stages [25–29]. These findings show a connexion between the opinion of pig farm and abattoir workers and those reported by experts. However, although they are aware of the underlying causes of animal welfare outcomes, many people in charge of caring for the animals and in charge of their welfare are hired caretakers and are not in a position to make changes or to adopt alternatives that could improve animal welfare. This can make them feel guilty when performing some painful procedures applied on farm (e.g., euthanasia [33]).

### Participant opinion

Most lay citizens considered that tail lesion, ear lesion and lameness are likely to cause suffering in pigs. Additionally, lay citizens attributed pigs with higher capacity to experience feelings and described them as being more intelligent and friendly than pig farm and abattoir workers. Other studies in Latin America have shown that people with links to the animal industries have different beliefs and attitudes regarding animal sentience and animal welfare compared to lay citizens, for example being more acceptant of controversial practices [34, 35]. However, our findings contrast those from Peden et al. [3], who found that British and Irish pig farmers showed similar perception regarding pig’s capacity to suffer than citizens unrelated to agriculture. Differences between studies may be related to the methodologies employed, for example questionnaires or interviews, and the questions asked. This is a relatively little explored issue and understanding the different views of different stakeholder requires more empirical studies comparing the groups.

Citizens prefer to eat animal product from production systems where the animals do not experience pain [36]. But although many citizens from different countries are concerned with animal suffering, as shown in a recent study with lay participants from 14 countries in different continents [37] and a growing proportion are becoming vegetarians due to moral concern for animals, including in developing countries [38], many others like to eat animal products. This has been discussed as “the meat paradox”, i.e. the psychological conflict between people moral response to animal suffering and their dietary preferences for and acceptance to eat meat [39]. Therefore, to support the sustainability of the meat industry, it is important to promote meat production systems that adopt practices favourable for animal welfare that have been scientifically validated and that, at the same time, is perceived by lay citizens as positive for animal welfare.

### Socio-demographic characteristics of participants

Female participants attributed pigs with higher capacity to experience emotions and had lower odds of agreeing that ear lesions are unlikely to cause suffering than male participants. This finding is in line with a previous study showing that women judge animal capacity to experience boredom and pain to be greater than men [3], and men are more likely to support castration without anaesthesia than women [40]. These findings were expected, as women are generally more concerned with animal welfare [41] and have greater empathy towards animals [42].

In our study, older participants had higher odds of agreeing that tail lesion and ear lesion are likely to cause suffering and to affect meat quality. This finding agrees with a previous survey reporting that older participants have greater belief in the mental capacities of animals [43], but contrasts with another, showing that younger people seem to be more concerned about animal welfare [3]. Regarding the effect of welfare outcomes on meat quality, lay citizens positively associated animal welfare outcomes with meat quality, which corroborates the findings of Teixeira et al. [44]. This concern is not unfounded regarding the case of tail lesions, as it is well known that this condition is associated with lower carcass weight and meat inspection outcomes [45–47]. Unfortunately, there is no study evaluating the association between ear lesion and meat quality in pigs.

### Effect of level of education

Lay citizens with completed or undergoing higher education described pigs as being more gluttonous, stubborn and dirty than participants with up to high school. University staff and students judged that pigs are more intelligent than other livestock species, and as intelligent as dogs and cats [48]. We did not ask our participants to specify their higher education course or area, but Peden et al. [3] reported that applied animal science students have higher perception on animals capacity to feel fear, pain and hunger than citizens unrelated to agriculture, probability because they have to learn about animal suffering during their degree studies.

It is important to highlight that these findings were based on a convenience sample of participants, especially from the pig industry, and thus do not represent the views of Chilean society or industry stakeholders. Therefore, we urge caution in interpreting our findings.

## Conclusion

We concluded that, compared to pig farm and abattoir workers recruited on farm and at the abattoir, lay citizens attributed pigs with higher capacity to experience feelings and described pigs as being more intelligent and friendly. The risk factors for tail lesion, ear lesions and lameness pointed out by pig farm and abattoir workers is in line with what have been suggested by experts. Also, most lay citizens considered that tail lesion, ear lesion and lameness are likely to cause suffering in pigs. Although we urge caution in interpreting our findings as it was based on a convenience sample of participants, our findings contribute to understand the perception and values of all stakeholders regarding animal welfare, as it is crucial to improve the sustainability of animal production systems.

## Supporting information

S1 File (PDF)

S2 File (PDF)
