# Supplementary material for: Pigs’ capacity to experience feelings and to suffer from tail lesion, ear lesion and lameness: Exploring citizens and pig farm and abattoir workers’ knowledge and perceptions
Source: PLoS One. 2023 May 25;18(5):e0286188. doi: 10.1371/journal.pone.0286188 (PMC10212169; doi:10.1371/journal.pone.0286188)
Supplement: S1 File — (PDF) [file pone.0286188.s001.pdf]

PONTIFICIA UNIVERSIDAD CATÓLICA DE CHILE

RESEARCH PROJECT

OPINION OF CHILEAN CITIZENS ON ISSUES RELATED TO PIG PRODUCTION SYSTEMS

Please, read each of the following questions carefully and mark your answer with an ‘x’.
<u>Please, make sure to answer all the questions</u>

Thank you for supporting our research! Date: ______________________

1. On a scale of 0 to 4, how much do you agree that pigs are able to feel the following feelings?

Completely disagree ... Completely agree

	0	1	2	3	4	I don’t know
Pain	○	○	○	○	○	○
Fear	○	○	○	○	○	○
Happiness	○	○	○	○	○	○
Anxiety	○	○	○	○	○	○
Boredom	○	○	○	○	○	○

2. On a scale of 0 to 4, how much do you agree with the following attributes when talking about pigs?

Completely disagree ... Completely agree

	0	1	2	3	4	I don’t know
Intelligent	○	○	○	○	○	○
Gluttonous	○	○	○	○	○	○
Friendly	○	○	○	○	○	○
Stubborn	○	○	○	○	○	○
Dirty	○	○	○	○	○	○

4. On a scale of 0 to 4, mark to what extent do you think the following injuries/conditions AFFECT THE TASTE, SMELL, COLOUR AND/OR APPEARANCE OF PIG MEAT OR ITS BY-PRODUCTS. (*Mark only **one option** for each injury/condition*)

	Unlikely 0	1	2	3	likely 4	I don't know
Tail lesions	○	○	○	○	○	○
Ear lesions	○	○	○	○	○	○
Lameness	○	○	○	○	○	○

5. Working place where you carry out your duties:

- ○ Pens and/or unloading
- ○ Finishing pig slaughter
- ○ Sow slaughter
- ○ Deboning
- ○ Transportation – truck driver
- ○ Administration/office
- ○ Laboratory
- ○ Other, please indicate below: ________________________

6. Sex

- ○ Male
- ○ Female

7. Age

- ○ 18-25
- ○ 26-35
- ○ 36-45
- ○ 46-55
- ○ over 56 years old

8. Education level

- ○ Up to high school
- ○ Higher education – completed or on-going

9. Do you have any kind of relationship with pig farming? ? (*Mark **only one option***)

- ○ **Not involved**
- ○ **Currently not,** but I grew up in an environment related to pig production
- ○ Yes, I am professionally involved in the pig industry (rural producer, student, academic, etc)

10. What kind of animal products do you consume?

- ○ Pork
- ○ Beef
- ○ Poultry meat
- ○ Fish
- ○ Milk and its by-products
- ○ Eggs
- ○ Sausages (salami, chorizo, etc.)
- ○ None

Thank you for supporting our research!
