# Supplementary material for: Pigs’ capacity to experience feelings and to suffer from tail lesion, ear lesion and lameness: Exploring citizens and pig farm and abattoir workers’ knowledge and perceptions
Source: PLoS One. 2023 May 25;18(5):e0286188. doi: 10.1371/journal.pone.0286188 (PMC10212169; doi:10.1371/journal.pone.0286188)
Supplement: S2 File — (PDF) [file pone.0286188.s002.pdf]

PONTIFICIA UNIVERSIDAD CATÓLICA DE CHILE

RESEARCH PROJECT

Opinion of personnel involved in pig production systems on issues related to animal husbandry

Please, read each of the following questions carefully and mark your answer with an 'x'.
Please, make sure to answer all the questions

Thank you for supporting our research! Date: ____________________

1. In the company or farm where you work, what do you think is the prevalence (number of cases) of the following injuries/conditions in animals?: (*Mark* ***only one option*** *for each injury/condition)*

	None (0%)	Very low (1 to 20%)	Low (21 to 40%)	Neither high or low (41 to 60%)	High (61 to 80%)	Very high (81 to 100%)	I don't know
Tail lesions	○	○	○	○	○	○	○
Ear lesions	○	○	○	○	○	○	○
Severe lameness	○	○	○	○	○	○	○

2. Please indicate the aspect(s) that you consider to be the main causes of the following injuries/conditions in animals:
(*For each injury/condition check at least one option*)

	Management	Lack of environmental enrichment	Use of antibiotics	Diet	Sex	Animal density	Floor type	Number of pens/building	Thermal insulation type	Pen orientation (North/South)	Pen location within building	Others	I do not know
**Tail lesions**	○	○	○	○	○	○	○	○	○	○	○	○	○
**Ear lesions**	○	○	○	○	○	○	○	○	○	○	○	○	○
**Lameness**	○	○	○	○	○	○	○	○	○	○	○	○	○

3. On a scale of 0 to 4, how much do you agree that pigs are able to feel the following feelings?

	Completely disagree 0	1	2	3	Completely agree 4	I don't know
Pain	○	○	○	○	○	○
Fear	○	○	○	○	○	○
Happiness	○	○	○	○	○	○
Anxiety	○	○	○	○	○	○
Boredom	○	○	○	○	○	○

4. On a scale of 0 to 4, how much do you agree with the following attributes when talking about pigs?

	Completely disagree 0	1	2	3	Completely agree 4	I don't know
Intelligent	○	○	○	○	○	○
Gluttonous	○	○	○	○	○	○
Friendly	○	○	○	○	○	○
Stubborn	○	○	○	○	○	○
Dirty	○	○	○	○	○	○

5. Working place where you carry out your duties:

- ○ Pens and/or unloading
- ○ Finishing pig slaughter
- ○ Sow slaughter
- ○ Deboning
- ○ Transportation – truck driver
- ○ Administration/office
- ○ Laboratory
- ○ Other, please indicate below:

____

6. Sex

- ○ Male
- ○ Female

7. Age

- ○ 18-25
- ○ 26-35
- ○ 36-45
- ○ 46-55
- ○ over 56 years old

8. Education level

- ○ Up to high school
- ○ Higher education – completed or on-going

Thank you for supporting our research!
